# Identification of genes and pathways involved in kidney renal clear cell carcinoma

**DOI:** 10.1186/1471-2105-15-S17-S2

**Published:** 2014-12-16

**Authors:** William Yang, Kenji Yoshigoe, Xiang Qin, Jun S Liu, Jack Y Yang, Andrzej Niemierko, Youping Deng, Yunlong Liu, A Keith Dunker, Zhongxue Chen, Liangjiang Wang, Dong Xu, Hamid R Arabnia, Weida Tong, Mary Qu Yang

**Affiliations:** 1 Department of Computer Science, George W. Donaghey College of Engineering and Information Technology, University of Arkansas at Little Rock, 2801 S. University Avenue, Little Rock, Arkansas 72204, USA; 2 Human Genome Sequencing Center, and Department of Molecular and Human Genetics, Baylor College of Medicine, Houston, Texas 77030, USA; 3 Department of Statistics, Harvard University, Cambridge, Massachusetts 02138, USA; 4 Division of Biostatistics and Biomathematics, Department of Radiation Oncology, Massachusetts General Hospital and Harvard Medical School, Boston, Massachusetts 02114, USA; 5 Rush University Cancer Center, and Departments of Internal Medicine and Biochemistry, Rush University Medical Center, Chicago, Illinois 60612, USA; 6 Center for Computational Biology and Bioinformatics, Indiana University School of Medicine, Indianapolis, Indiana 46202, USA; 7 Department of Epidemiology and Biostatistics, Indiana University School of Public Health, 1025 E. 7th Street, PH C104, Bloomington, Indiana 47405, USA; 8 Department of Genetics and Biochemistry, Clemson University, Clemson, South Carolina 29634, USA; 9 Department of Computer Science, University of Missouri, Columbia, Missouri 65211, USA; 10 Department of Computer Science, University of Georgia, Athens, Georgia 30602, USA; 11 Divisions of Bioinformatics and Biostatistics, National Center for Toxicological Research, United States Food and Drug Administration, 3900 NCTR Road, Jefferson, Arkansas 72079, USA; 12 MidSouth Bioinformatics Center, Department of Information Science, George W. Donaghey College of Engineering and Information Technology, University of Arkansas at Little Rock, 2801 S. University Avenue, Little Rock, Arkansas 72204, USA; 13 Joint Bioinformatics Graduate Program, University of Arkansas at Little Rock and University of Arkansas for Medical Sciences, Little Rock, Arkansas 72204, USA

**Keywords:** Kidney Renal Clear Cell Carcinoma, TCGA, RNA-Seq, Differentially Expressed Genes, Pathways, Gene Network Analysis, Machine Learning Classifier

## Abstract

**Background:**

Kidney Renal Clear Cell Carcinoma (KIRC) is one of fatal genitourinary diseases and accounts for most malignant kidney tumours. KIRC has been shown resistance to radiotherapy and chemotherapy. Like many types of cancers, there is no curative treatment for metastatic KIRC. Using advanced sequencing technologies, The Cancer Genome Atlas (TCGA) project of NIH/NCI-NHGRI has produced large-scale sequencing data, which provide unprecedented opportunities to reveal new molecular mechanisms of cancer. We combined differentially expressed genes, pathways and network analyses to gain new insights into the underlying molecular mechanisms of the disease development.

**Results:**

Followed by the experimental design for obtaining significant genes and pathways, comprehensive analysis of 537 KIRC patients' sequencing data provided by TCGA was performed. Differentially expressed genes were obtained from the RNA-Seq data. Pathway and network analyses were performed. We identified 186 differentially expressed genes with significant *p-value *and large fold changes (P < 0.01, |*log*(FC)| > 5). The study not only confirmed a number of identified differentially expressed genes in literature reports, but also provided new findings. We performed hierarchical clustering analysis utilizing the whole genome-wide gene expressions and differentially expressed genes that were identified in this study. We revealed distinct groups of differentially expressed genes that can aid to the identification of subtypes of the cancer. The hierarchical clustering analysis based on gene expression profile and differentially expressed genes suggested four subtypes of the cancer. We found enriched distinct Gene Ontology (GO) terms associated with these groups of genes. Based on these findings, we built a support vector machine based supervised-learning classifier to predict unknown samples, and the classifier achieved high accuracy and robust classification results. In addition, we identified a number of pathways (P < 0.04) that were significantly influenced by the disease. We found that some of the identified pathways have been implicated in cancers from literatures, while others have not been reported in the cancer before. The network analysis leads to the identification of significantly disrupted pathways and associated genes involved in the disease development. Furthermore, this study can provide a viable alternative in identifying effective drug targets.

**Conclusions:**

Our study identified a set of differentially expressed genes and pathways in kidney renal clear cell carcinoma, and represents a comprehensive computational approach to analysis large-scale next-generation sequencing data. The pathway and network analyses suggested that information from distinctly expressed genes can be utilized in the identification of aberrant upstream regulators. Identification of distinctly expressed genes and altered pathways are important in effective biomarker identification for early cancer diagnosis and treatment planning. Combining differentially expressed genes with pathway and network analyses using intelligent computational approaches provide an unprecedented opportunity to identify upstream disease causal genes and effective drug targets.

## Background

Cancer is not only complex, in that many genetic variations can contribute to malignant transformation, but also wildly heterogeneous, in that genetic mechanisms can vary between patients of same pathological type. Kidney Renal Clear Cell Carcinoma (KIRC) is the eighth most common cancer and is known to be the most lethal of all the genitourinary tumours with an estimation of approximately 65,000 new cases and approximately 13,000 deaths annually in United States [1]. This disease is known resistant to radiotherapy and chemotherapy [2], and there are very few cases that have been reported to respond immunotherapy [3]. If KIRC can be detected in very early stages, it is potentially curable by surgical resection, while adjuvant therapies have not been proven beneficial. The recurrence rate is not very high, although still considered not uncommon. Nevertheless, there is no curative treatment for late stage KIRC. The 2-year survival rate of patients with metastatic KIRC is less than 20% [4,5]. Therefore, further investigations of the genomic alterations and underlying molecular mechanisms are essential for early diagnosis and treatment. As cancer is a consequence of the accumulation of genetic alterations and dysregulation of pathways, identification of differentially expressed genes and pathways is important. We aimed to develop integrative approaches to identify differentially expressed genes and pathways in combination with gene network analysis for finding effective early cancer biomarkers and drug targets.

In this study, we designed computational approaches to identify differentially expressed genes from the RNA-seq data provided by the TCGA data portal. We further performed Gene Ontology (GO) analysis and categorized expression patterns. Categorization of differentially expressed genes and expression patterns suggested distinct disease subtypes that are associated with distinct biological processes. Many studies have indicated that same type of cancer can have different subtypes with different genetic mechanisms and treatment responses. Bannon et.al. discovered two distinct KIRC subtypes using gene microarray expression data [6], whereas four stable subtypes of KIRC were detected using both mRNA and miRNA expression data sets [7]. Despite of discoveries of differentially expressed genes and genetic mutations, the knowledge of biological pathways involved in the disease is limited. To facilitate the effective biomarker identification, we therefore further analysed pathways and gene networks related to the differentially expressed genes.

KEGG (Kyoto Encyclopaedia of Genes and Genomes, http://www.genome.jp/kegg/) pathway analysis revealed that differentially expressed genes are significantly enriched in a number of biological pathways that are known in cancer, as well as previously unreported pathways. This study provided new insights into the regulatory mechanisms of KIRC through comprehensive differential gene expression, pathway and network analyses.

## Results

### Differentially expressed genes in KIRC

**Figure 1:**
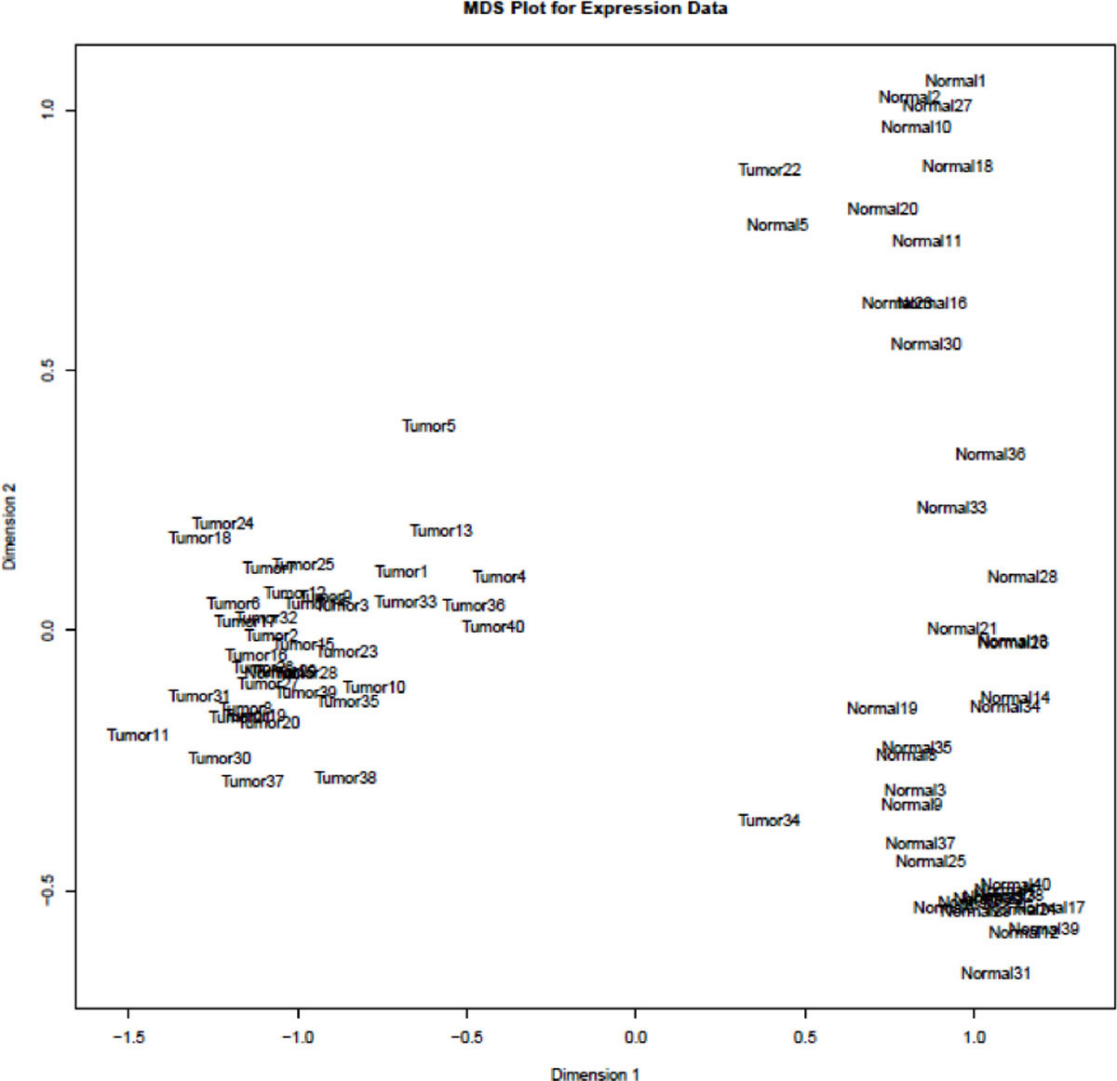
**MDS plot for RNA-seq gene expression of KIRC tissue and normal tissue samples**. Tumour refers to tumour tissue samples from KIRC patients. Normal refers to the matched normal tissue samples from the same patient. There are totally 68 tumour and 68 paired normal tissue samples in the MDS plot.

**Figure 2:**
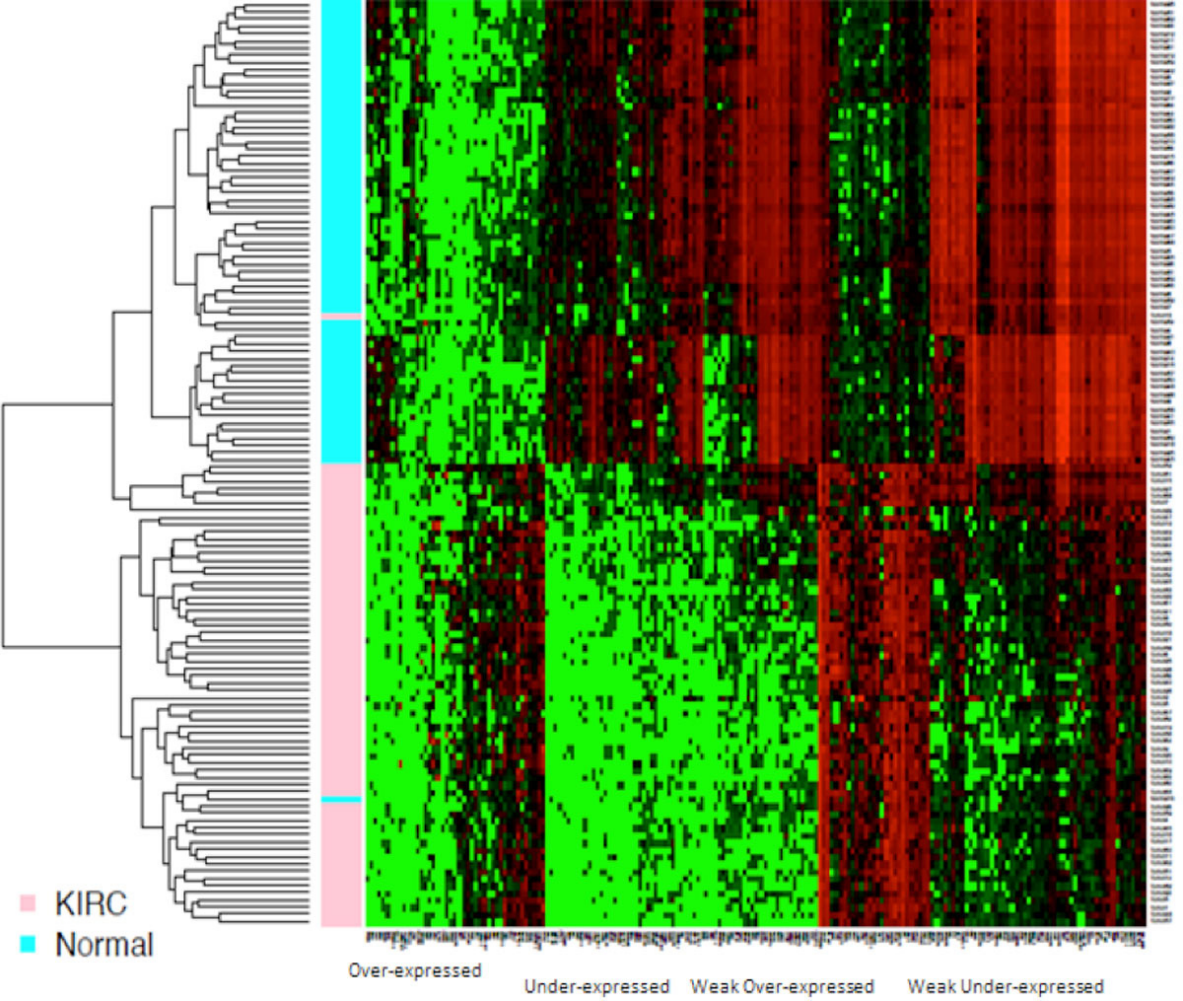
**Hierarchical clustering of 136 KIRC and normal tissue samples based on the expression level of the differential genes**. The figure shows hierarchical clusters of tumour and normal tissues using expression levels of 186 differential genes. In the column sidebar of the figure, pink represents KIRC tissue samples and cyan represents normal tissue samples. In the heatmap, green represents genes that are down-regulated whereas red represents genes that are down-regulated.

**Figure 3:**
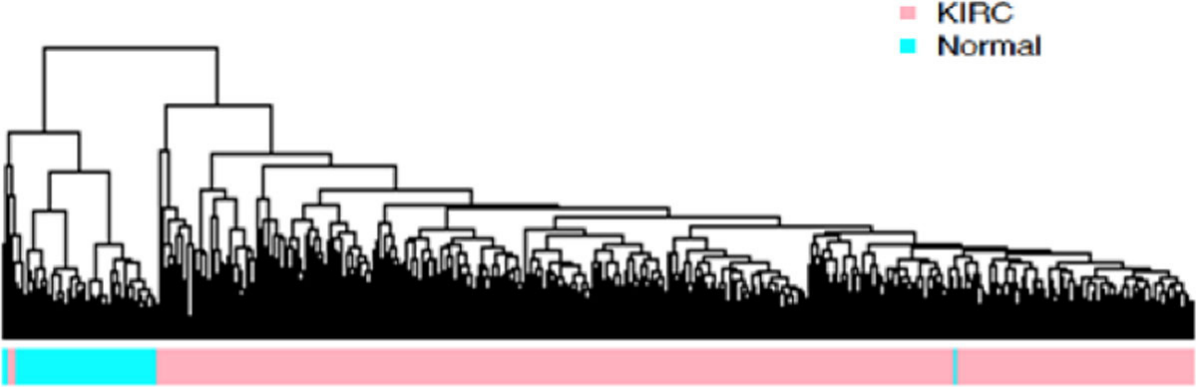
**Clustering of 537 KIRC and normal tissue samples based on the expression level of the differential genes**. The figure shows hierarchical clusters of all samples including matched and unmatched samples. The KIRC tumour samples were seperated well from normal samples by using expression levels of differential genes revealed in matched samples.

Analysis of 186 differentially expressed genes was performed using RNA-seq data of 537 KIRC patients from the TCGA data portal, including both matched and unmatched samples. Matched samples have sequencing data from both normal kidney and kidney cancer tissues in the same KIRC patients, whereas unmatched samples only have sequencing data from either disease or no disease tissues. TCGA data not only contain 68 matched KIRC tumour and normal kidney tissue paired sequencing data, but also provide 469 unpaired tumour and 4 normal kidney tissue sequencing data. We applied multidimensional scaling (MDS) on the 68 paired KIRC samples and found that almost all tumour and normal samples were separated well from each other (Figure 1). The MDS result suggested that TCGA data quality was acceptable. We performed differential gene expression analysis using edgeR [8,9] package. Using statistical P-values and fold changes (FC) as selection criteria, we identified 186 differentially expressed genes with P < 0.01, and |*log*(FC)| > 5 (additional file 1). These differentially expressed genes not only confirmed some previous research findings but also provided new findings. For example, *TNFAIP*, a known tumour *α*-induced protein that acts as a natural brake on inflammation [10], was up-regulated in KIRC in the analysis. Another differentially expressed gene, *SLC6A3*, which is implicated in lung and breast cancer [11,12], has not yet been well known in KIRC. Based on the expression levels of differentially expressed genes, we performed hierarchical clustering analysis and revealed homogenous tumour and normal (no cancer) clusters (Figure 2). Only one normal sample and one tumour sample were mis-clustered, but both samples came from the same patient (paired samples). This suggested that they are more likely mislabelled rather outliers. The expression levels of the differential genes showed distinct patterns between tumour and normal kidney tissue samples. We clustered both overly expressed genes and genes with diminished expression in tumour samples (Figure 2). We performed GO (Gene Ontology) [13] analysis on four groups of genes defined as over-expressed, under-expressed, weakly over-expressed and weakly under-expressed. Fisher exact test and Benjamini-Hochberg multiple testing corrections were performed to obtain significance with GO terms. The significant GO terms associated with each group are listed in Table 1. The over-expressed genes are significantly associated with biological processes of defence responses and responses to environmental stimulus. The under-expressed genes are associated with organismal physiological processes, system development and organ development. Furthermore, we performed hierarchical clustering of all samples including 469 unpaired KIRC and 4 normal samples using the differentially expressed genes detected from the matched samples. In consistence with the results of the matched sample analysis, we obtained two homogeneous clusters; only 4 including the mis-labelled sample out of all 537 samples (Figure 3) were mis-clustered, demonstrating that overall, the identified differentially expressed genes can effectively distinguish KIRC from normal kidney tissue samples. Furthermore, we have combined the pathway and network analyses to enhance overall classification performance. From the comprehensive analyses, the up-regulated genes were strongly associated with cellular responses to the tumour development, and the down-regulated genes implicated compromises of kidney function as result of the cancer. Kidney functional damages are common among cancer patients. In addition to biological function association, the distinct gene groups lead to the identification of subtypes of KIRC.

**Table 1:** Biological functions associated with distinct differential gene groups.

Gene Group	GO term (P < 0.01, Fisher's Exact Test with Benjamini multiple test correction)
Over-expressed	Defence response Response to environmental stimulus

Under-expressed	Organismal physiological process System development Organ development

Weakly over-expressed	Cell-cell signalling Lip metabolism Signal transmission across a synapse Organismal physiological process

Weakly under-expressed	Excretion Secretion Transmembrane transported activity

### Biological pathways associated with KIRC

**Figure 4:**
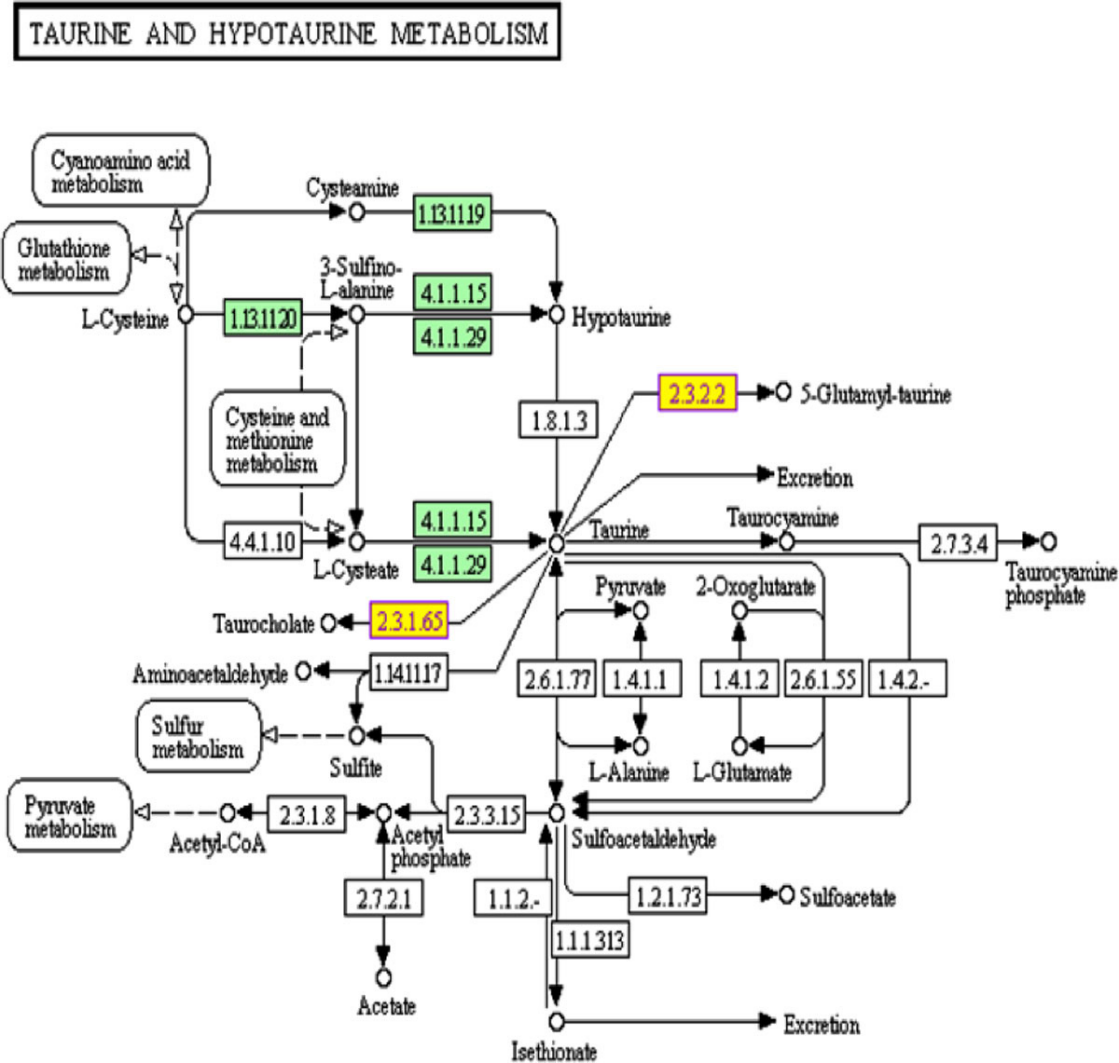
**The taurine and hypotaurine metabolism pathway**. This KEGG pathway is enriched for differential genes, colored with yellow, in KIRC.

**Figure 5:**
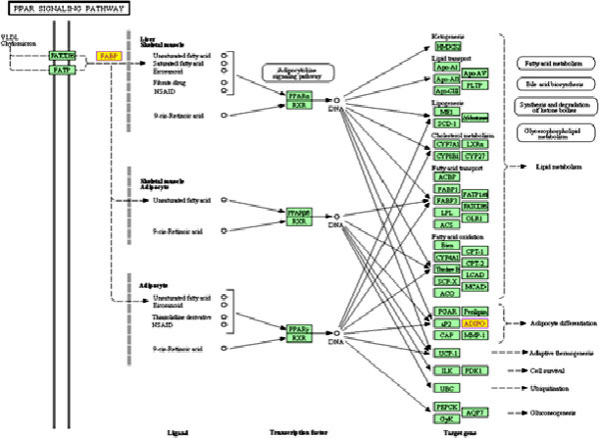
**The PPAR signalling pathway**. This KEGG pathway is enriched for differential genes, colored with yellow, in KIRC.

**Figure 6:**
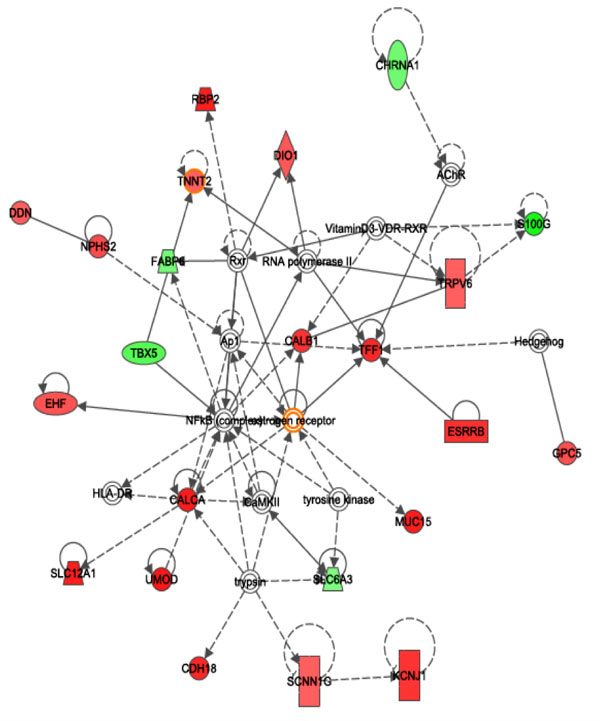
**Network of molecular transport, heredirary disorder, metabolic disease**. In the figure, over-expressed genes were marked in red and under-expressed genes were marked in green. The node(s) circled with yellow represent gene(s) that can be used as biomarker for the disease.

**Figure 7:**
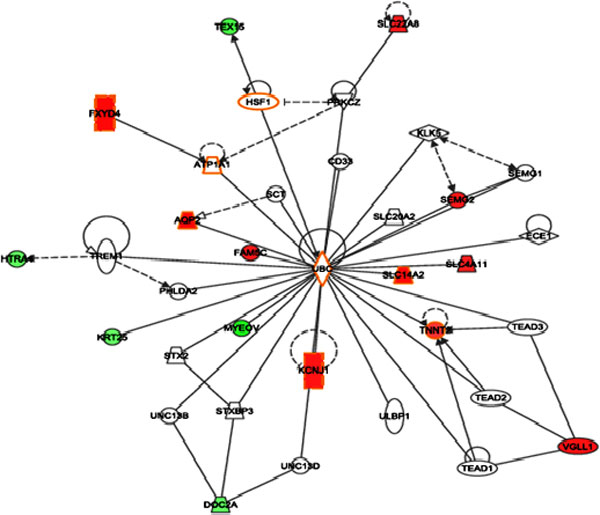
**Network of renal and urological disease**. In the figure, over-expressed genes were marked in red and under-expressed genes were marked in green. The node(s) circled with yellow represent gene(s)s that can be used as biomarker for the disease.

We employed hypergeometric test to assess the differentially expressed genes in KEGG [14] pathway analysis to identify significant pathways involved in the cancer. Significantly affected pathways in KIRC include taurine and hypotaurine metabolism (Figure 4), neuroactive ligand-receptor interaction, glycosaminoglycan biosynthesis - heparin sulphate, Peroxisome Proliferator-Activated Receptor (PPAR) signalling pathway (Figure 5), and hepatitis C, gastric acid secretion pathway (Table 2). Methylation of the genes in taurine and hypotaurine metabolism has been associated with the worst prognosis in renal cell carcinoma [15]. Hepatitis C pathway has been implicated in kidney tumour [16-18]. Some of those pathways are known related to cancer, for example, PPAR signalling pathway has been shown related to renal cell carcinoma [19]. In addition, Ingenuity (http://www.ingenuity.com) pathway analysis revealed most significant networks (P < 0.01), including network of molecular transport, hereditary disorder, metabolic disease (Figure 6), and network of renal and urological disease (Figure 7), respectively. In the figures, nodes in red colour represent genes that are up-regulated, while notes in green colour represent genes that are down-regulated in the disease. Nodes circled in yellow colour represent genes that have been known implicated in cancer. Interestingly, most differentially expressed genes located at the periphery of the networks are more likely downstream targets rather than regulators themselves. The central node of molecular transport, hereditary disorder, and metabolic disease network is *NF-κB *(nuclear factor kappa-light-chain-enhancer of activated B cells), which is known active in kidney carcinoma and plays roles in tumour development as well as therapeutic resistance [20,21]. *UBC (ubiquitin C)*, a gene associated with protein degradation, DNA repair and cell cycle regulation, is located at the centre of the renal and urological disease network, and interconnects many other genes in the network as a hub protein. *UBC *has been implicated in breast cancer and contributes to cancer metastasis [22]. In cancer studies, we often firstly identify differentially expressed genes to help find the biomarkers for tumour early detection, and then use the results to identify upstream disease causal genes through further network analysis. In this study, we combined the investigation of differentially expressed genes with pathway and network analyses. Combining gene expression profiles with pathway and network analyses has helped the identification of regulators for downstream genes, regardless that the expression levels were significantly altered or not.

**Table 2:** KEGG pathways that are significantly enriched for differential genes.

P-value (hypergeometric test)	Pathway
0.002	Taurine and hypotaurine metabolism

0.016	Neuroactive ligand-receptor interaction

0.025	Glycosaminoglycan biosynthesis - heparin sulfate

0.033	Peroxisome proliferator-activated receptor (PPAR) signalling pathway

0.0346	Hepatitis C

0.039	Gastric acid secretion

### Construction of a classifier for KIRC prediction

We built a predictive classifier using Support Vector Machine (SVM) algorithm to classify unknown tissue samples. The expression levels of differential genes were utilized as input features. To achieve robust performance, we have used boosting with bootstrapping and aggregation algorithm and randomly selected 4/5 of samples for training, while the remaining 1/5 of samples were used for testing. We repeated the process for 50 times. Sensitivity and specificity were used to assess the classifier performance. As there is always a trade-off between specificity and sensitivity, we used Receiver Operating Characteristic (ROC) curve to measure the overall performance of the classifier. If the area under ROC curve is 1.0 or 100%, it means the classifier is perfect. The SVM-based classifier achieved average sensitivity, specificity and area under ROC curve from 50 trials as 96.5%, 97.0% and 98.7% respectively. The performances of the classifier were summarized in Table 3. The results demonstrated that the classifier has achieved accurate and robust classification performance with low standard deviations. The intelligent machine can be used successfully for categorizing disease samples with high accuracy.

**Table 3:** Performance of the classifier

SVM-based Classifier	Sensitivity	Specificity	Area under ROC
Mean	96.5%	97.0%	98.7%

Standard deviation	0.036	0.036	0.015

## Discussion

For identification of differentially expressed genes, we used 68 paired tumour and normal kidney tissue samples. In each paired samples, one sample was from KIRC tumour tissue and the paired sample was from the pathologically normal kidney tissue from the same patient. Using paired sequencing data obtained from the same patient can reduce false positives due to genetic difference or other confounding factors that are not necessarily related to the cancer. This is particularly advantageous in avoiding genetically natural polymorphisms among the population. Although only the matched sets of samples were used in the identification of differentially expressed genes, we have obtained highly homogenous clusters using expression levels of differentially expressed genes in clustering all samples including 469 unmatched KIRC and 4 normal kidney tissue samples. Moreover, the SVM-based classifier utilizing the expression levels of differentially expressed genes was shown capable of predicting unknown tissue samples with high accuracy, suggesting that the differentially expressed genes can serve as expression signatures of the disease. Analyses of gene expression profiles and differently expressed genes suggested four different subtypes of the cancer. This was further supported by the pathway and network analyses that have revealed differentially disturbed pathways and networks influenced by the subtypes of the disease. Analyses of cancer related pathways and networks not only confirmed some of discoveries that have been already reported in literature, but also provided new findings. Amongst the significant networks that we identified, *NF-κB *and *UBC *are examples of central nodes for molecular transport, hereditary disorder, metabolic disease network and network of renal or urological disease. Interestingly, the expression levels of both *NF-κB *and *UBC *were not significantly altered in KIRC, instead they highly interconnect with other genes in the network structure, suggesting their important regulatory roles in the cancer. Though they can be considered as markers of the disease, the differentially expressed gene set shall be further utilized to infer upstream disease causal genes by combining pathway and network analyses with the systems biology approaches. This research was part of investigations of integrative systems biology approaches to identify disrupted pathways in disease development (http://www.world-academy-of-science.org/worldcomp14/ws/keynotes/invited_talk_yang). Integrating the disease-associated networks with differentially expressed genes and pathways can lead to the identification of useful biomarkers and effective drug targets.

## Conclusions

Our differentially expressed genes and pathway analyses have utilized large-scale RNA-seq data and have provided new insights into the molecular mechanisms in the cancer. In the study, the expression levels of differentially expressed genes detected in the paired tumour samples were used as input features for the machine learning classifier. We were able to identify a set of genes associated with molecular perturbations in the disease development, and obtain highly homogenous clusters. The intelligent machine built for this study was able to achieve high accuracy in clustering and classifying the tumour samples. Indeed, pathway and network analyses based on significant genes not only confirmed pathways implicated in the disease, but also identified new roles of unreported pathways in the cancer. Combing differentially expressed genes with pathway and network analyses not only provided an unprecedented opportunity to reveal subtypes of the disease, but also better understanding of underlying molecular mechanisms related the cancer development.

## Methods

### Differentially expressed genes in KIRC

The RNA-seq and meta-data of KIRC were downloaded from TCGA Data Portal Bulk Download (https://tcga-data.nci.nih.gov/tcga). The availability of more than 500 cancer patient data has been used advantageously to train the high performance classifier. Tumour purification and data quality were investigated. All samples contained at least 60% tumour nuclei by pathological determination [7]. Since there are common concerns of sample impurity in cancer genomics analysis, the next-generation sequences from this quality of cancerous tissue samples were considered as sufficient. RNA-seq version 2 data were provided by University of North Carolina Genome Centre using the RNA-seq data protocol generated by the Illumina HiSeq. Normal tissue samples were defined as pathologically no cancerous nuclei and micronuclei, and the normal tissue samples contained 4 tissues from healthy (no cancer) human kidneys, and 68 tissues from either paired pathologically normal portions of the disease kidney or paired other side no-cancer healthy kidneys from the KIRC patients. The cancerous tissue samples included 68 matched and 469 unmatched tumour tissue samples. All cancerous tissue samples contained about 2/3 or more tumorous cell nuclei pathologically. The edgeR package [8,9] was used for differentially expressed gene identification. Support Vector Machine was designed for classifying KIRC samples.

### Gene ontology and pathway analyses

We performed Gene Ontology (GO) analysis on differentially expressed genes in KIRC. Fisher exact test with multiple test correction was used to obtain significant GO terms that are associated with differentially expressed genes. We also searched for enrichment of differentially expressed genes in KEGG pathway analysis. Hypergeometric test was applied to attain significant KEGG pathways. GO and pathway analyses were conducted using Bioconductor packages [23]. In addition, Ingenuity Pathway Analysis (http://www.ingenuity.com) was applied to identify differential networks in KIRC.

### Machine learning classifier

Using expression levels of the differentially expressed genes, SVM-based classifier was designed to distinguish cancer from no cancer samples. Different kernels were tested and linear kernel was adopted in the SVM model. Boosting with Bootstrapping Aggregation algorithm was designed. We used five-fold cross-validation to assess the performance of the classifier. Receiver operating characteristic (ROC) curves were generated. The performance of the machine was evaluated using sensitivity and specificity defined as:

$$\text{Sensitivity}=\frac{TP}{TP+FN}$$

$$\text{Specificity}=\frac{TN}{TN+FP}$$

where TP is the count of true positives, here referring to number of true cancer samples; *TN *is the count of true negatives, referring to number of non-cancer (normal tissue) samples; *FP *is count of the false positives, referring to non-cancer tissue samples that were misclassified as cancer; and *FN *is the count of false negatives, referring to cancer tissue samples that were misclassified as normal (no-cancer) tissue samples. Since there is always a trade-off between specificity and sensitivity, the area under ROC curves were used to evaluate the overall performance of the classifier. A high performance classifier can reach up to 1.0 (100%) for area under ROC while a random classifier has just about or barely above 0.5 (50%) on the area under ROC. The SVM-based classifier achieved average 96.5% sensitivity, 97.0% specificity and 98.7% of area under ROC respectively, thus the classifier can effectively identify cancer samples.

## Competing interests

The authors declare that they have no competing interests.

## Authors' contributions

MQY conceived and designed the project. WY implemented the project, wrote scripts, software artefacts, and data mining tools. KY, XQ, JSL, JYY, AN, YD, YL, AKD, ZC, LW, DX, HRA, and WT provided expertise, guidance and participated in the study and discussions. WY performed the experiment, analysed the results and drafted the manuscript. WY and MQY revised and finalized the paper which was read and approved by all authors.

## Supplementary Material

Additional File 1 List of 186 differentially expressed genes and *log *(FC) ( |*log *(FC)| > 5). Click here for file

## Declaration

The funding for publication of the article has come from the MidSouth Bioinformatics Centre, and the Joint Bioinformatics Ph.D. Program of University of Arkansas at Little Rock and University of Arkansas for Medical Sciences with NIH/NIGMS 5P20GM10342913 and ASTA award # 15-B-23.

This article has been published as part of *BMC Bioinformatics *Volume 15 Supplement 17, 2014: Selected articles from the 2014 International Conference on Bioinformatics and Computational Biology. The full contents of the supplement are available online at http://www.biomedcentral.com/bmcbioinformatics/supplements/15/S17.
